# Structural Insights into the Dual Strategy of Recognition by Peptidoglycan Recognition Protein, PGRP-S: Structure of the Ternary Complex of PGRP-S with Lipopolysaccharide and Stearic Acid

**DOI:** 10.1371/journal.pone.0053756

**Published:** 2013-01-09

**Authors:** Pradeep Sharma, Divya Dube, Mau Sinha, Savita Yadav, Punit Kaur, Sujata Sharma, Tej P. Singh

**Affiliations:** Department of Biophysics, All India Institute of Medical Sciences, New Delhi, India; Consejo Superior de Investigaciones Cientificas, Spain

## Abstract

Peptidoglycan recognition proteins (PGRPs) are part of the innate immune system. The 19 kDa Short PGRP (PGRP-S) is one of the four mammalian PGRPs. The concentration of PGRP-S in camel (CPGRP-S) has been shown to increase considerably during mastitis. The structure of CPGRP-S consists of four protein molecules designated as A, B, C and D forming stable intermolecular contacts, A–B and C–D. The A–B and C–D interfaces are located on the opposite sides of the same monomer leading to the the formation of a linear chain with alternating A–B and C–D contacts. Two ligand binding sites, one at C–D contact and another at A–B contact have been observed. CPGRP-S binds to the components of bacterial cell wall molecules such as lipopolysaccharide (LPS), lipoteichoic acid (LTA), and peptidoglycan (PGN) from both Gram-positive and Gram-negative bacteria. It also binds to fatty acids including mycolic acid of the *Mycobacterium tuberculosis* (*Mtb*). Previous structural studies of binary complexes of CPGRP-S with LPS and stearic acid (SA) have shown that LPS binds to CPGRP-S at C–D contact (Site-1) while SA binds to it at the A–B contact (Site-2). The binding studies using surface plasmon resonance showed that LPS and SA bound to CPGRP-S in the presence of each other. The structure determination of the ternary complex showed that LPS and SA bound to CPGRP-S at Site-1 and Site-2 respectively. LPS formed 13 hydrogen bonds and 159 van der Waals contacts (distances ≤4.2 Å) while SA formed 56 van der Waals contacts. The ELISA test showed that increased levels of productions of pro-inflammatory cytokines TNF-α and IFN-γ due to LPS and SA decreased considerably upon the addition of CPGRP-S.

## Introduction

The 19 kDa peptidoglycan recognition protein (PGRP-S) is one of the four mammalian PGRPs which were originally classified according to their molecular weights as PGRP-S (M.W., 20–25 kDa), PGRP-Iα and PGRP-Iβ (M.W., 40–45 kDa) and PGRP-L (M.W. up to 90 kDa) [1]. PGRP-S has been detected in bone marrow [2] and granules of polymorphonuclear leucocytes [2]. It is also found in the mammary secretions [3] as well as in the intestinal M cells [4]. The significant concentration of PGRP-S has so far been reported in the mammary secretions of camel (*Camelus dromedarius*) only [3]. As part of the innate immune system, mammary PGRP-S contributes to the protection of animal udder as well as to the new borns against the invading microbes. It recognizes various pathogen-associated molecular patterns (PAMPs) with high affinity [1]. We have shown that the components of bacterial cell wall molecules such as lipopolysaccharide (LPS) of Gram-negative bacteria, lipoteichoic acid (LTA) of Gram-positive bacteria and peptidoglycans (PGNs) of both Gram-positive and Gram-negative bacteria as well as mycolic acid (MA) and other fatty acids of the *Mycobacterium tuberculosis* [5]–[7] bind to camel PGRP-S (CPGRP-S) with affinities ranging from micromolar to nanomolar [8]–[10]. The structural studies have shown that CPGRP-S adopts a unique quaternary structure with four molecules, A, B, C and D forming two stable interfaces one between molecules A and B (A–B contact) and the second between molecules C and D (C–D contact) [8]–[10]). The A–B and C–D interfaces involve two opposite faces of a monomer leading to the formation of the linear chain with alternating A–B and C–D contacts. The previous studies have shown that LPS, LTA and PGN bind to CPGRP-S at Site-1 which is situated at the C–D contact while mycolic acid and other fatty acids were held at Site-2 at the A–B contact [9]–[12]. Having obtained these results, it was pertinent to determine whether CPGRP-S could bind to the components of multiple bacterial cell wall molecules simultaneously through its two independent binding sites or it would bind to only one kind of PAMPs at a time. Therefore, the binding studies of CPGRP-S with LPS and SA were carried out in the presence of each other which showed that LPS and SA bound to CPGRP-S with similar affinities as those reported in the bimolecular interactions [9]. In order to reveal the mode of binding of two different types of cell wall molecules simultaneously, a ternary complex of CPGRP-S with LPS and SA was crystallized. The structure determination of the complex showed that LPS and SA were observed bound to Site-1 and Site-2 respectively. This indicated the binding potential of CPGRP-S to interact with two independent PAMPs through its two separate binding sites, S-1 and S-2.

## Materials and Methods

### Ethics Statement

The peripheral blood was taken from the healthy human volunteers with the approval of the Institute Ethics Committee at All India Institute of Medical Sciences, New Delhi, India. The written consent had been given by the donors before blood samples were collected from them.

### Purification

Fresh samples of camel milk were obtained from the National Research Centre on Camels, Bikaner, India. The skimmed milk was diluted twice with 50 mM Tris-HCl pH 8.0. The cation exchanger CM-sephadex (C-50), pre-equilibrated with 50 mM Tris-HCl pH 8.0 at a concentration of 7 g/l was added to the diluted samples and stirred slowly for 1 hour with a glass rod. The gel was allowed to settle for half an hour after which the solution was decanted. The gel was washed with excess of 50 mM Tris-HCl, pH 8.0. It was packed in a column (25×2.5 cm) and washed with same buffer until the absorbance reduced to 0.05 at 280 nm. After this, the bound basic proteins were eluted with 0.5 M NaCl in 50 mM Tris-HCl pH 8.0 and desalted by dialyzing it against triple distilled water. The desalted fraction was again passed through a CM-sephadex (C-50) column (10×2.5 cm) which was pre-equilibrated with 50 mM Tris-HCl pH 8.0 and eluted with 0.05–0.5 M NaCl in the same buffer. The eluted fractions were examined on the sodium dodecyl sulphate polyacrylamide gel electrophoresis (SDS-PAGE). The fractions corresponding to a molecular weight of approximately 20 kDa were pooled. The pooled fractions were concentrated using Amicon ultrafiltration cell. The concentrated protein was passed through Sephadex G-100 column (100×2 cm) using 50 mM Tris-HCl pH 8.0. Two peaks were obtained when the fractions were read at 280 nm wavelength. The purity of the eluted fractions was checked using SDS-PAGE which indicated that the second peak in the gel filtration profile corresponded to the molecular weight of 20 kDa of PGRP-S. The high molecular weight first peak also showed a band at about 20 kDa on the SDS-PAGE indicating a polymeric state of CPGRP-S.

### Binding Studies

Freshly purified sample of CPGRP-S was immobilized on a CM5 carboxyldextran chip using carbodiimide chemistry to a level of 10000 response units (RU) as described previously (9, 10) using a BIAcore-T200 (BIAcore). In one experiment three different concentrations (200 nM, 150 nM, and 100 nM) of analytes, SA and LPS were passed over the immobilized CPGRP-S at a flow rate of 10 µl/min with an injection time of 420 seconds. The regeneration of bound analytes was done using 10 mM NaOH for 100 seconds at a flow rate of 30 µl/min. The association (k_on_) and dissociation constants (k_off_) for the binding of analytes to CPGRP-S were calculated and values of equilibrium constants were obtained using mass action relation K_D_ = k_off_/k_on_ with BIA evaluation software provided by the manufacturer. In another experiment only binding analysis of both the analytes was done by passing both the analytes one after the other with injection time of 420 seconds and flow rate of 10 µl/min. However, the regeneration procedure was carried only once at the end of experiment. The resulted sensogram was recorded which showed that both analytes bound to CPGRP-S in the presence of each other.

### Induction of TNF-α and IFN-γ by LPS and SA

The peripheral blood mononuclear cells (PBMCs) were isolated from heparinized blood by Ficoll Hypaque gradient centrifugation and suspended in complete RPMI-1640 with 10% fetal calf serum (FCS) at optimum culture conditions of 5% CO_2_, at 37°C for 6 hrs. Cells were stimulated with medium alone and with 10 µg/ml LPS and SA without and with 10 µg/ml CPGRP-S. The culture supernatants were collected after 6 hours of stimulation at optimum culture conditions and assayed for TNF-α and IFN-γ concentrations by ELISA according to manufacturer’s instructions. The data were expressed as mean values ± standard deviations. The statistical differences in the results were evaluated by student’s t-test.

### Crystallization

Freshly purified samples of protein were dissolved in the buffer containing 50 mM Tris-HCl pH 8.0 to a concentration of 15 mg/ml. The 10 µl protein solution was mixed with an equal volume of the reservoir solution containing 10% polyethylene glycol-3350 (PEG-3350) and 0.2 M sodium potassium tartrate. This mixture was vortexed for 5 minutes to make it homogenous. The 10 µl drops were set up in the hanging drop vapour diffusion method against the above reservoir solution. The crystals grew to approximate dimensions of 0.4×0.3×0.3 mm^3^ in about two weeks. The freshly grown crystals were soaked for more than 48 hours in the solution containing 70% reservoir solution and 30% ethanol into which LPS and SA were dissolved at 20 mg/ml concentration. These soaked crystals were used for X-ray intensity data collection.

### X-ray Intensity Data Collection and Processing

Crystals of CPGRP-S were stabilized by the addition of 30% glycerol for data collection at low temperature. A single crystal was mounted in a nylon loop and flash-frozen in liquid nitrogen at 100 K. A complete data set was collected using the DBT-sponsored MX beamline, BM14 at ESRF, Grenoble, France with a wavelength of, λ = 0.98 Å on 165 mm MAR CCD detector (MAR RESEARCH, Norderstedt, Germany). The data were processed with AUTOMAR and SCALEPACK from HKL package [13]. The results of data collection are given in Table 1.

**Table 1:** Data collection and refinement statistics for the structure of the ternary complex of CPGRP-S with LPS and SA.

	PGRP-S+LPS+SA
PDB ID	4 GF9
Space group	I222
Unit cell dimensions	
a (Å)	89.6
b (Å)	101.9
c (Å)	162.3
Number of molecules in theasymmetric unit	4
Vm (Å^3^/Da)	2.32
Solvent Content (%)	47.0
Resolution range (Å)	50.0–2.8
Number of unique reflections	37206
#Rsym (%)	7.9 (28.9)
I/σ(I)	33.9 (4.3)
Overall completeness of data (%)	98.8 (99.5)
*R_cryst_ (%)	22.9
R_free_ (%)	26.6
Protein atoms	5348
Water oxygen atoms	256
Atoms of LPS	48
Atoms of SA	20
Atoms of glycerol	6
R.m.s.d in bond lengths (Å)	0.02
R.m.s.d in bond angles (°)	1.9
R.m.s.d in torsion angles (°)	18.0
Mean B-factor for main chain atoms (Å^2^)	11.4
Mean B-factor for side chain atoms (Å^2^)	12.7
Mean B-factor for all atoms(Å^2^)	12.1
Ramachandran’s φ, ψ map	
Residues in the most favoured regions (%)	90.2
Residues in the additionally allowedregions (%)	9.8

The values in parentheses correspond to the values in the highest resolution shell.

# R_sym_  =  ∑_hkl_∑_i_ | I_i_(hkl) – <I(hkl)> |/∑_hkl_ ∑_i_ I_i_(hkl).

* R_cryst_  =  ∑_hkl_ | F_o_(hkl) − F_c_(hkl) |/∑_hkl_ | F_o_(hkl) | where F_o_ and F_c_ are observed and calculated structure factors respectively.

### Structure Determination and Refinement

**Figure 1:**
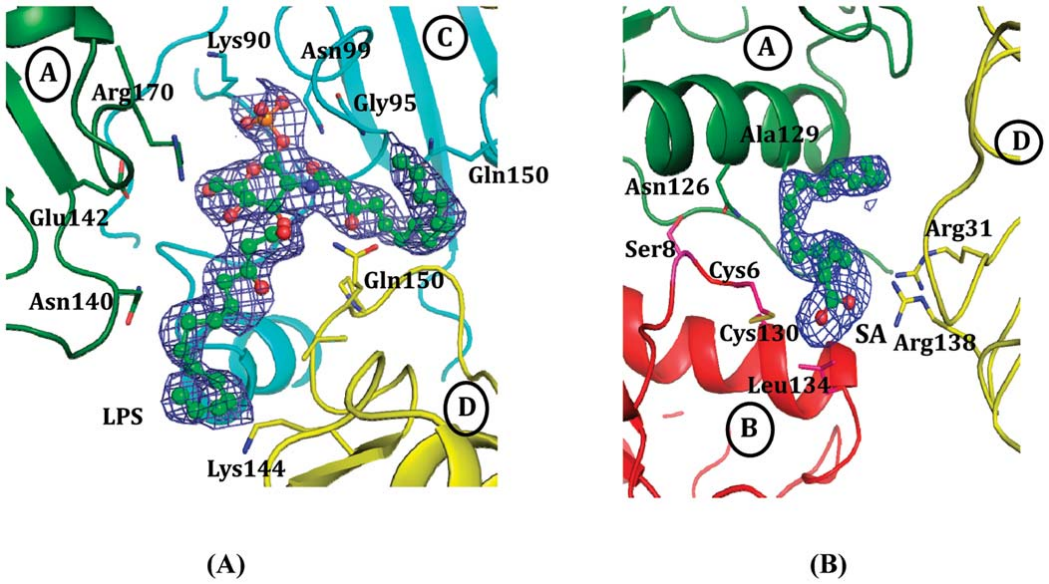
Initial difference Fourier map (Fo−Fc) contoured at 2.0 σ for (A) SA and (B) LPS.

The structure of the ternary complex of CPGRP-S formed with LPS and SA was refined using the structure of native CPGRP-S (PDB Code: 3C2X) (8) as the starting model. The structure consisted of four crystallographically independent protein molecules which were designated as A, B, C and D. The refinement for the data to 2.8 Å resolution was carried out with program REFMAC 5.5 [14]. The model was improved by repeated manual model buildings using program O [15] and Coot [16]. The tight main-chain and side-chain non-crystallographic symmetry restraints between the four molecules were used in the refinement. The electron density maps (2Fo−Fc) and (Fo−Fc) were calculated to adjust the protein chain in the electron density. After several rounds of model rebuilding and intermittent cycles of refinement, R_cryst_ factor dropped to 0.282. The group temperature factor (B) refinement was used with further model adjustments yielding R_cryst_ factor of 26.3%. The difference Fourier (Fo−Fc) map computed at this stage revealed additional non-protein but quite characteristic electron densities at 2σ cutoffs at two sites which were located at the C–D and A–B contacts. LPS molecule was fitted into the electron density on Site-1 at the C–D contact while SA was fitted in Site-2 at A–B contact (Figure 1). The coordinates of atoms of both ligands were added to the model in the further cycles of refinement with isotropic B-factors. At this stage, the positions of 256 water oxygen atoms were also obtained from the difference Fourier map. These were added in the subsequent cycles of refinement. The water oxygen atoms were removed from the model if they were closer than 2.3 Å from the nearest atom. They were also removed if they were farther than 3.5 Å or if the electron densities at these locations fell below 2.5 σ. The refinement converged with values of final R_cryst_ and R_free_ factors of 22.9% and 26.6% respectively. As indicated by calculations using program PROCHECK [17], 90.2% residues were found in the most favoured regions of the Ramachandran’s φ, ψ map [18] while 9.8% residues were found in the additionally allowed regions. The details of refinement parameters are given in Table 1.

## Results

### Binding Analysis

**Figure 2:**
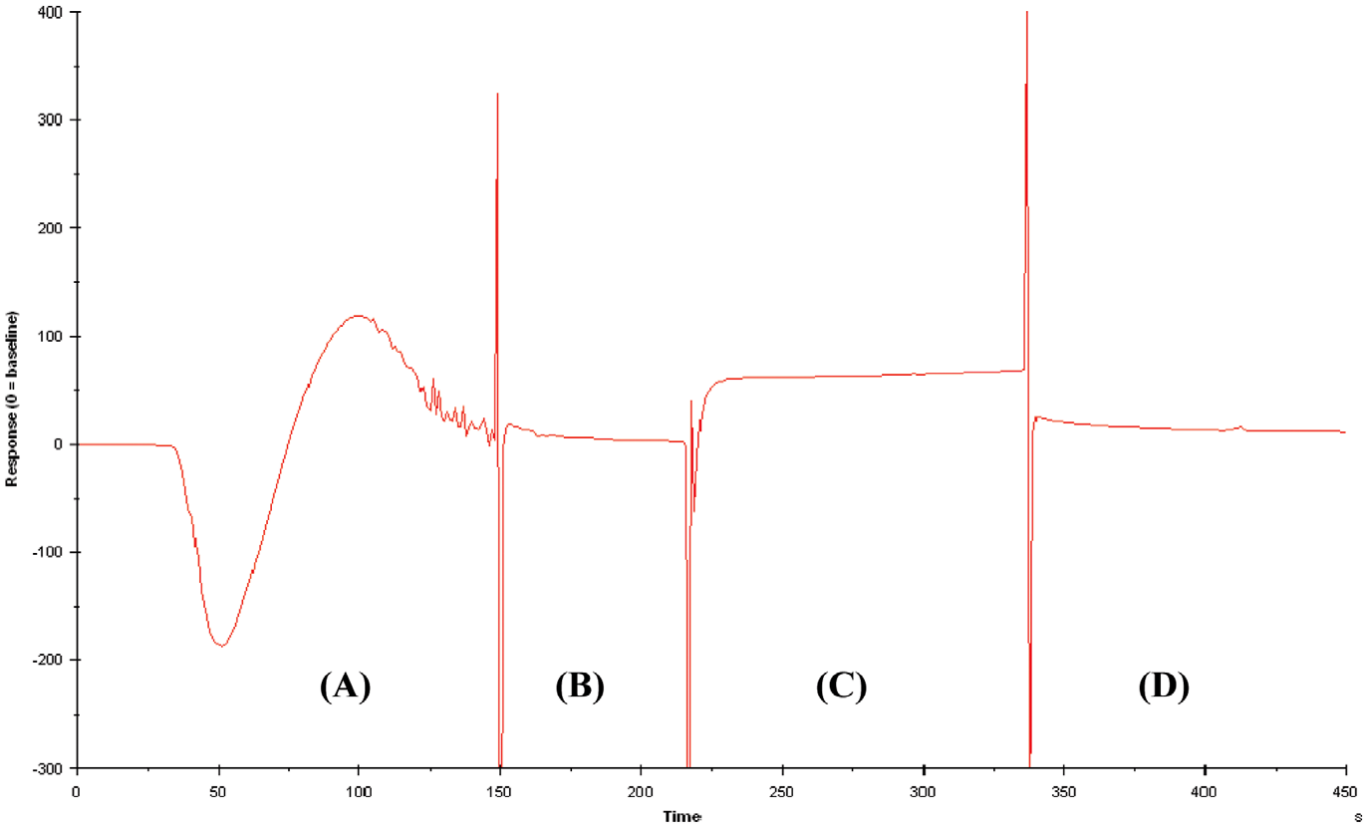
Sensogram for the binding of (A) LPS and (C) SA. (B) and (D) regions corresponding to injection stage.

The binding studies of CPGRP-S using SPR were carried out with both ligands, LPS and SA. It has been shown by previous structural studies of binary complexes of CPGRP-S with LPS and SA [9]–[11] that LPS bound to CPGRP-S in the binding Site-1 at the C–D contact while SA was found to bind the protein in the binding Site-2 at the A–B contact [19]. Since the two binding sites were located distantly from each other, the surface plasmon resonance studies were carried out with both ligands separately as well as one after the other. As the protein was immobilized on the chip, LPS was injected onto it at a flow rate of 10 µl/min. It showed binding with final RU of 108. Then SA was injected to the LPS-bound protein at the same flow rate. It showed binding with final RU of 76. The binding experiment was also carried out in the reverse order which also showed similar RU values. As seen from the sensogram (Figure 2) both compounds bound to the protein. Since the bindings of SA to LPS-bound protein as well as that of LPS to SA-bound protein occurred, the formation of ternary complex was clearly established.

### Inhibition of LPS and SA Induced Expressions of TNF-α and IFN-γ

**Figure 3:**
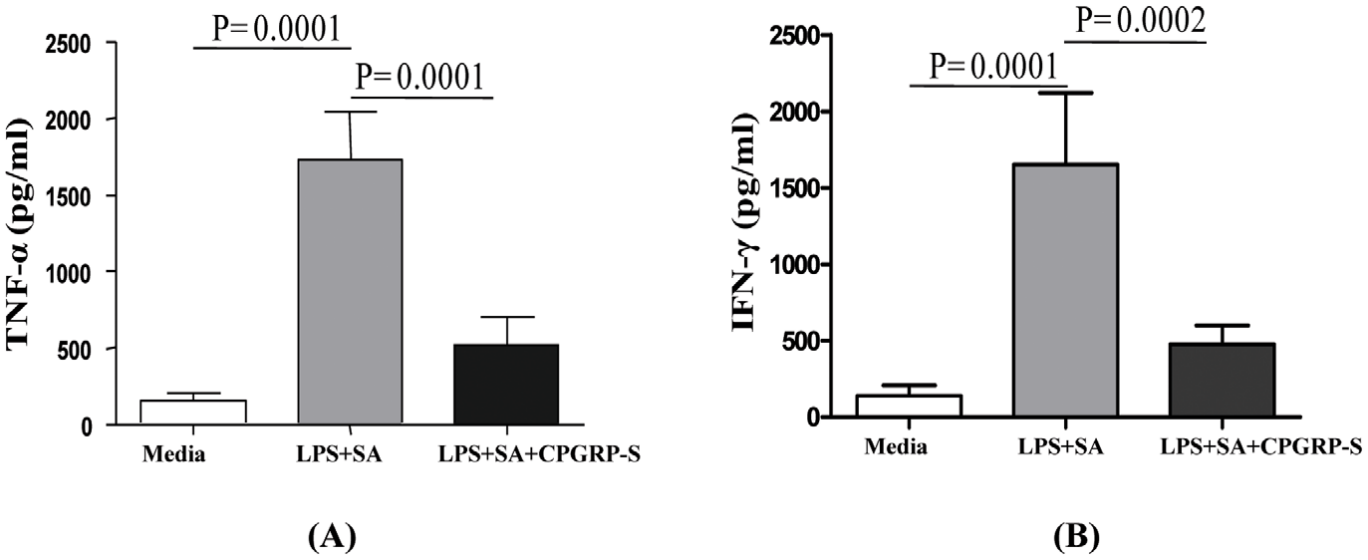
Inhibition of LPS+SA induced pro-inflammatory cytokines, TNF-α and IFN-γ when CPGRP-S was added to the medium along with LPS and SA.

The recognition of LPS by immune cells is a significant component of the acute adaptive and memory immune response. The critical indicators of the pathogenesis of bacterial infection are the copious amount of production of pro-inflammatory cytokines TNF-α and IFN-γ predominantly by macrophages and T cells. In order to determine the efficiency of CPGRP-S to inhibit the production of pro-inflammatory cytokines such as TNF-α and IFN-γ, the cultured PBMCs were challenged with the mixture of LPS and SA and the observed pro-inflammatory cytokines were assayed in the cultured PBMCs. The treatment of PBMCs with 10 µg/ml of LPS and SA mixture increased the production of TNF-α and IFN-γ by 6.2 and 7.5 folds respectively (Figure 3) in comparison to media alone. The increased levels of TNF-α and IFN-γ were almost completely abolished when the cells were incubated with 10 µg/ml of LPS and SA mixture along with 5 µg/ml of CPGRP-S. This indicated that CPGRP-S inhibited the pro-inflammatory effects of LPS and SA.

### Overall Structure

**Figure 4:**
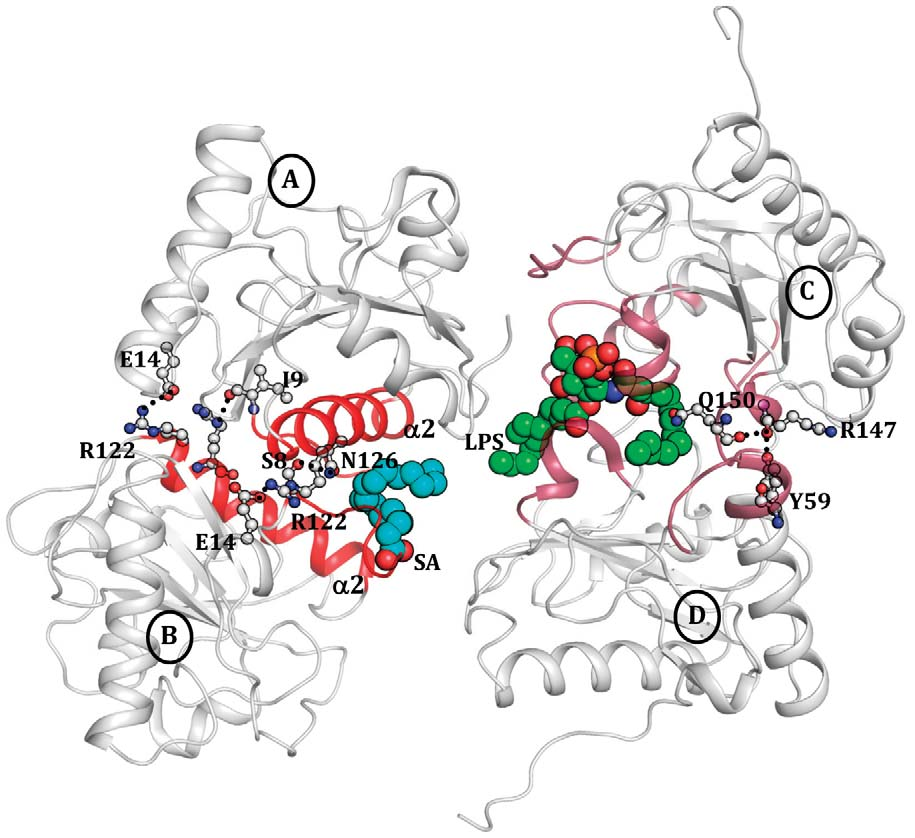
Structure of the ternary complex of CPGRP-S with LPS and LTA. The binding sites are shown in different colours. SA and LPS are shown as space fitting models in blue and green colours respectively.

**Figure 5:**
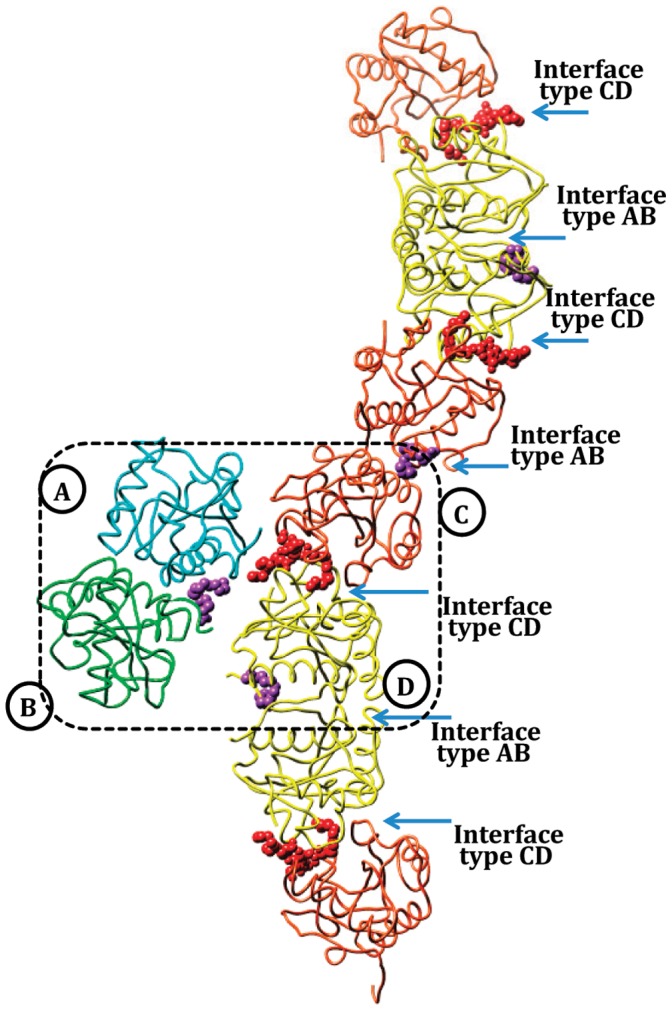
View of the structure of the ternary complex of CPGRP-S showing four crystallographically independent molecules in the asymmetric unit which is indicated by dashed lines. CPGRP-S molecules assemble as a linear polymer forming A–B and C–D contacts alternatingly. The bound molecules of SA at Site-2 and LPS at Site-1 are also shown as space filling models.

Crystal structure of CPGRP-S consists of four crystallographically independent molecules A, B, C and D in the asymmetric unit in associations as A–B and C–D dimers (Figure 4). An examination of the intermolecular interactions of the packing of molecules in the crystal together with the buried surface areas between them indicated that A–B interface provided the most stable association with an approximate buried surface area of 798 Å^2^ while C–D interface was slightly less stable with a buried surface area of 702 Å^2^. The A–C and B–D interfaces were found to be weakly associated with buried surface areas of 340 Å^2^ and 111 Å^2^ respectively. A further examination of the packing of molecules in the crystal revealed that the interface between molecule D and its symmetry related molecule D′ and also molecule C and its symmetry related molecule C′ showed identical buried areas as that of A–B interface. In fact, it represented the same contact as represented by A and B monomers. It showed that one surface of monomer formed A–B interface while its opposite surface was part of the C–D interface. Both these surfaces of the monomer are located on opposite sides of the monomer. Thus the structure of CPGRP-S can be described as a contiguous chain of protein molecules in which A–B and C–D contacts occur alternatingly (Figure 5). The molecular mass of the first peak in the elution profile obtained using size exclusion chromatography was estimated based on the void volume. This value was similar to that determined by extrapolating the value of hydrodynamic radii observed using dynamic light scattering of the protein [19]. These values were similar to that derived from the polymeric nature of the structure as indicated by the structure determination [19]. The previous structural studies have shown that there are two independent ligand binding sites. One site is located at the A–B contact while the other is situated at C–D contact. In the present structure, the binding site at the A–B contact is occupied by SA while the one at C–D contact is filled by LPS. It may be mentioned here that the structures of all the four protein molecules are identical with rms deviations of less than 0.6 Å for their C^α^ positions. The overall structure of CPGRP-S monomer consists of a central β-sheet with five β-strands, β3 (residues, 31–38), β4 (residues, 71–76), β5 (residues, 80–85), β6 (residues, 103–108) and β7 (residues, 142–146). The α/β structure of the protein consists of three main α-helices, α1 (residues, 46–64), α2 (residues, 118–134) and α3 (residues, 157–164). The α-helices α2 of molecule A (Aα2) and molecule B (Bα2) are part of A–B interface while loops Tyr59 - Trp66, Ala94 - Asn99 and Arg147 - Leu153 are from molecules C and D as part of the C–D interface. The binding cleft at the A–B contact is formed with the help of helices Aα2 and Bα2 together with N-terminal segments, Glu1– Glu14 of molecule A(AN) and molecule B(BN).

### Structure of A–B Contact and Interactions of SA

**Figure 6:**
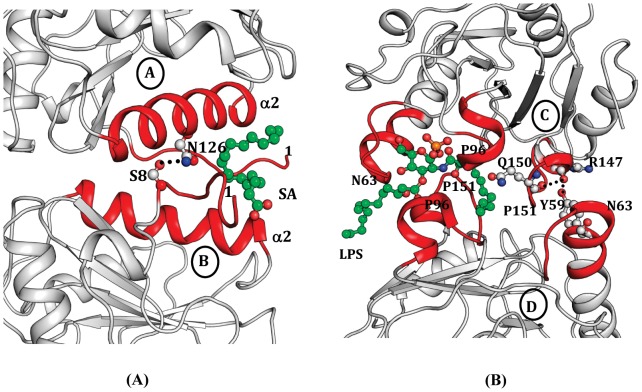
The binding of SA and LPS to CPGRP-S, (A) A section of A–B contact showing a bound SA molecule in the cleft. The binding is essentially stabilized by van der Waals contacts. (B) A section of C–D contact showing a bound LPS molecule in the cleft. The binding is stabilized by several hydrogen bonds and a network of van der Waals contacts.

The interface between molecules A and B represents an extended buried region with a buried surface area of approximately 800 Å^2^. The interface consists of α-helix α2 (residues, 118–134), N-terminal segment (residues, 1–14), loops (residues, 43–49) and (residues, 76–81) from each molecule (Figure 4). The important residues that form hydrogen bonds/ionic interactions and stabilize the A–B interface include Ser-8, Ile-9, Glu-14, Arg-122 and Asn-126. The hydrogen bonds, Ser-8(A)N••••••Asn-126(B)O^δ1^ = 3.2 Å, Ser-8(A)O^γ^••••••Asn-126(B)O^δ^  = 2.6 Å are critical for dimerization and unique to CPGRP-S as the corresponding residues in HPGRP-S are Pro and Gly respectively. Overall, the intermolecular interactions between molecules A and B include 8 hydrogen bonds/salt bridges and 90 van der Waals contacts (distances ≤4.2 Å). The ligand binding cleft at the A–B contact is formed involving α-helices, Aα2 and Bα2 as well as N-terminal segments AN and BN. The helices, Aα2 and Bα2 are inclined at an angle of 45° with each other with a wider opening on the outer side towards the surface of the dimer. Therefore, the arrangement of the helices Aα2 and Bα2 at the interface creates a funnel-like structure with a narrow end on the inner side and wider opening on the outer side towards the surface of the A–B interface (Figure 6A). The interior of these two amphipathic α2 helices contain a series of hydrophobic residues. The flexible N-terminal segments AN and BN are hooked to the funnel with the help of disulfide linkages, Cys-6A••••••Cys-130A and Cys-6B••••••Cys-130B. SA is placed in the cleft at the A–B contact and it forms atleast 48 van der Waals contacts with amino acid residues, Asn-126, Ala-129, Val-132 and Ala-133 from molecule A and Pro-4, Ala-5, Cys-6, Ala-133 and Leu-134 from molecule B. These interactions of SA in the ternary complex were approximately similar to those observed in its binary complex. However, the number of interactions involving SA was considerably larger than those reported for butyric acid, lauric acid, myristic acid as well as those of the fragment of mycolic acid [19]. Nevertheless, the most common interactions involving Cys-6 from molecule B were identical in both the binary and the ternary complexes. The other three residues, Ala-129 from molecule A and Ala-133 and Leu-134 from molecule B were found interacting in both binary and ternary complexes of SA with CPGRP-S. Therefore, the mode of binding of SA to CPGRP-S is very similar in both binary and ternary complexes indicating that the binding site at A–B contact is not perturbed by the binding of ligands at the C–D contact.

### Structure of C–D Contact and Interactions with LPS

The C–D interface is formed with two monomers through surfaces opposite to that of A–B contacts. The buried surface area at this interface was found to be of the order of 702 Å^2^. This association is stabilized by six intermolecular hydrogen bonds and 74 van der Waals contacts (distances ≤4.2 Å). This interface consists of three important loops with residues, Tyr-59− Trp-66, Ala-94− Asn-99 and Arg-147− Leu-153 from each molecule. The important residues at the interface are four prolines, Pro-96C, Pro-151C, Pro-96D and Pro-151D. It may be noted here that the corresponding residues in HPGRP-S are His-99 and Arg-154 which are considered to be unfavorable for intermolecular stacking. This ligand binding cleft is situated at one end of the C–D interface having a glycan binding pocket inserted in molecule C. This ligand binding cleft consists of amino acid residues, Ser-20, Thr-27, Trp-66, Asp-68, Arg-85 and Asn-99 from molecule C and a segment Gly-91– Asn-99 from molecule D (Figure 6B). Upon interaction with CPGRP-S, the glycan moiety of LPS is hooked into the glycan binding pocket in molecule C while the two hydrocarbon chains extend into two different directions whereby one is pushed into the binding space at the interface while the other one is aligned along the outer surface of molecule D. As a result of this several contacts are made by LPS with molecules C and D to produce a stable complex. The LPS molecule makes extensive contacts with protein molecules C and D with atleast two dozens of hydrogen bonds and a large number of van der Waals contacts. The residues that are involved in hydrogen bonded interactions are Trp-66, Arg-85, Lys-90, Gly-91, Ala-92, His-93 and Asn-99 from molecule C while residues, Thr-97, Asp-98, Val-149 and Gln-150 are from molecule D. These interactions of LPS in the ternary complex are similar to those reported in the binary complex [9]. The positions and interactions of residues Lys-90 and Asn-99 were identical in both structures indicating the significance of their roles in the recognition of LPS.

## Discussion

So far, crystal structures of PGRP-S have been determined from two species that include human [20] and camel [8]. Although the molecular structures of human (HPGRP-S) and camel (CPGRP-S) proteins are similar with rms deviations of 0.8 Å for C^α^-positions, their quaternary structures are completely different. The polypeptide chain of human PGRP-S consisting of residues from 9–175 was found to adopt the monomeric state [20] while the full chain (residues, 1–171) CPGRP-S was observed in a polymeric state [12]. The crystal structure determination of CPGRP-S showed that it consisted of four crystallographically independent molecules A, B, C and D in the asymmetric unit which is arranged in a linear chain with alternating A–B and C–D contacts (Figure 5). In such an arrangement, two ligand binding sites were observed. These are situated at the sites of A–B and C–D contacts (Figures 6). In case of HPGRP-S, the corresponding surfaces of the monomer may also act as binding sites. Indeed one of the monomeric surfaces has been shown to bind to PGN [10] while the opposite face to it was assumed to bind to non-PGN types of effector molecules [20]. The comparison of amino acid sequences of CPGRP-S and HPGRP-S [9] shows the presence of several residues in the sequence of CPGRP-S on the two surfaces of the monomer that have been reported to be favorable for dimerization of proteins [8]. These residues are Ala-5, Gly-7, Ser-8 and Asn-126 at the A–B interface and Ilu-89, Lys-90, Ala-94, Pro-96, Tyr-97, Pro-151 and Arg-158 at the C–D interface. Similarly, the binding cleft at the A–B contact has a favourable stereochemistry for the binding of fatty acids indicating a possibility for the recognition of cell wall molecules including mycolic acid of the *Mycobacterium tuberculosis*. The cleft at the C–D contact has been shown to be involved in the binding of cell wall molecules of bacteria other than *Mycobacterium tuberculosis*. These molecules included LPS, LTA and PGN of Gram-negative and Gram-positive bacteria [10]. The structure of the ternary complex of CPGRP-S with LPS and SA provides another strong evidence of the recognition potential of CPGRP-S for acting against bacterial infection. The observed forcep-like shape of the cleft formed by two α-helices Aα2 and Bα2 at the A–B contact provides features similar as that observed in the case of other fatty acid binding proteins [21], [22]. On the other hand the cleft at the C–D contact consists of a specific pocket for the recognition of glycan moieties such as GlcNAc and MurNAc [11]. In a contrast, it was shown in the structures of the complexes of PGRP-S domain of HPGRP-Iα and HPGRP-Iβ, that the peptide moiety of PGN was the initial element of recognition by the protein [23], [24]. Therefore, the real issue here was whether the specificity pocket at the C–D contact was more suitable for binding to glycan components of PGNs or it suited more to bind to the interlinking peptide Thus it important to understand as to which of the two moieties played a more significant role in the recognition of PGNs by PGRP-S. Since glycan moieties are the conserved chemical entities of bacterial cell wall molecules these might be preferred elements for the recognition. This has been shown by several structures of the complexes of CPGRP-S with various PAMPs [9]–[12], [19]. On the other hand, the peptide sequences in PGNs vary considerably and may require a very promiscous peptide recognition site. Also, the peptide components in PGNs interconnect the glycan chains and hence they might not be fully accessible for specific recognition by the protein. In view of these facts and also as observed in the structures of the complexes of CPGRP-S with various PAMPs, the glycan moieties indeed appeared to be more relevant elements for the recognition by CPGRP-S at the C–D contact.

An examination of intermolecular interactions between CPGRP-S and SA and between CPGRP-S and LPS clearly showed that both ligands bound to the protein strongly and independently. As there is no plausible site in CPGRP-S for enzymatic activity, the binding appears to be the only mode of action. Thus CPGRP-S may sequester bacteria and deprive it of cell-cell communication as well as it may prevent the bacterial contact with the matrix around it. Such an isolation of bacterial cells may eventually cause its death. This process of bacterial killing here appears to be different from that of antibacterial peptides such as defensins that kill bacteria by permeabilization of cell membranes [25], peptidoglycan lytic enzymes which also kill bacteria by causing membrane permeabilization [26]. However, it may have some similarity with the action of antibiotics such as penicillin that may eventually destroy the cell wall of bacteria by inhibiting its synthesis [27]. Thus, the kinetics of bacterial killing by CPGRP-S may be somewhat similar to that of antibiotics and because of this similarity CPGRP-S may also be termed as a protein antibiotic.
